# Quantifying Airborne Lidar Bathymetry Quality-Control Measures: A Case Study in Frio River, Texas

**DOI:** 10.3390/s18124153

**Published:** 2018-11-27

**Authors:** Kutalmis Saylam, John R. Hupp, John R. Andrews, Aaron R. Averett, Anders J. Knudby

**Affiliations:** 1 Near Surface Observatory, Bureau of Economic Geology, John A. and Katherine G. Jackson School of Geosciences, The University of Texas at Austin, Austin, TX 78758, USA; John.Hupp@beg.utexas.edu (J.R.H.); John.Andrews@beg.utexas.edu (J.R.A.); Aaron.Averett@beg.utexas.edu (A.R.A.); 2 Shallow Water Earth Observation Laboratory, Department of Geography, Environment and Geomatics, The University of Ottawa/Université d’Ottawa, Ottawa, ON K1N 6N5, Canada; aknudby@uottawa.ca

**Keywords:** Lidar bathymetry, environmental conditions, uncertainties, quality management

## Abstract

Airborne Lidar Bathymetry (ALB) is an advanced and effective technology for mapping water bodies and measuring water depth in relatively shallow inland and coastal zones. The concept of using light beams to detect and traverse water bodies has been around since the 1960s; however, its popularity has increased significantly in recent years with the advent of relatively affordable hardware, supplemented with potent software applications to process and analyze resulting data. To achieve the most accurate final product, which is usually a digital elevation model (DEM) of the bottom of a water body, various quality-control (QC) measures are applied during and after an airborne mission. River surveys, in particular, present various challenges, and quantifying the quality of the end product requires supplemental surveys and careful analysis of all data sets. In this article, we discuss a recent ALB survey of the Frio River in Texas and summarize the findings of all QC measures conducted. We conclude the article with suggestions for successful ALB deployments at similar survey locations.

## 1. Introduction

**Figure 1:**
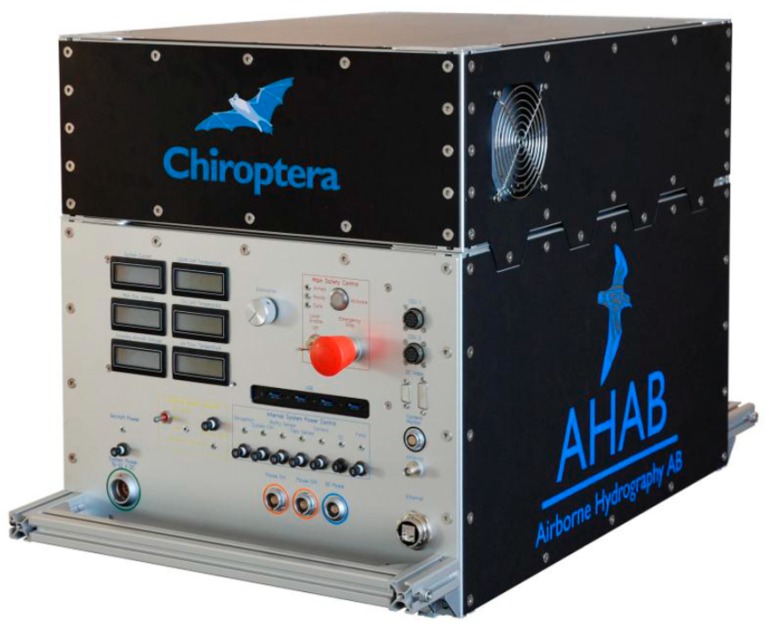
Custom-built Leica AHAB Chiroptera.

**Figure 2:**
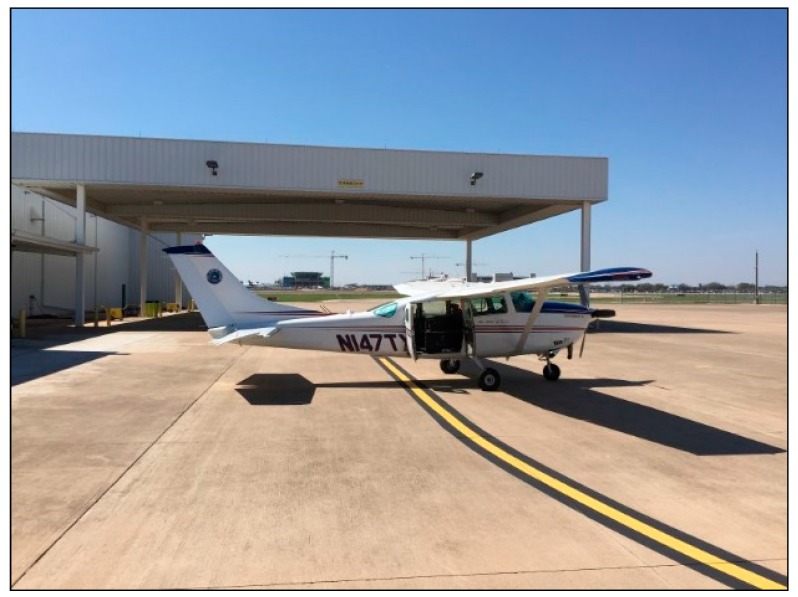
AHAB Chiroptera was installed in a Texas Department of Transportation (TxDOT) owned and operated Cessna 206 aircraft.

Airborne Lidar Bathymetry (ALB) is a technology for characterizing the depths of shallow-water bodies in relatively transparent waters from an airborne platform using a scanning and pulsed light beam. The Bureau of Economic Geology (the Bureau) at The University of Texas at Austin owns and operates a Leica AHAB “Chiroptera” ALB system, and has access to multiple survey aircraft with varying mission capabilities (Figure 1 and Figure 2). For data collection, Chiroptera uses a near-infrared (NIR) wavelength (1064 nm) for topographic and a green wavelength (515 nm) for bathymetric. The effective range is 400 to 500 m for the bathymetric scanner, which acquires data with a continuous waveform signal. The topographic scanner enables surveys up to 1500 m above ground level and can record pulses up to 400 kHz at lower altitudes. Both scanners direct the light beam at a fixed incident angle to 14° at fore and aft, and 20° to each side, creating an elliptical pattern on the surface (Palmer scanner). Advantages of this elliptical scanning pattern include its capability to map sloped surfaces such as cliffs and building facades, and to measure the terrain underneath moderate tree canopies. For visual-analysis purposes, Chiroptera integrates a 50-megapixel Hasselblad medium-format camera, which acquires imagery simultaneously with Lidar data. At ALB survey altitudes, ground-sampling distance of an image is approximately 6 cm, producing fine details of the survey location features.

In recent years, use of ALB systems for hydrographic surveying of shallow waters has proved to be a versatile, cost-effective, and detailed method compared to other remote-sensing technologies such as sonar and satellite imaging [1,2]. The Bureau has been involved in airborne Lidar surveys since 2000 [3,4] and has completed hundreds of ALB survey hours, covering areas larger than 9000 km^2^ at diverse locations and conditions. For all projects, specific quality-management and quality-control (QC) methods were applied to achieve the most consistent and precise final products [5]. QC practices take place during and after the data acquisition phase to ensure that final products provide the desired level of quality. For ALB surveys, QC practices involve routine in situ checks and supplementary surveys with other available technologies such as sonar and GPS. Because the quality of the Lidar-derived surface models relies heavily on the accuracy of the Lidar system components, it is essential to have accurate calibration parameters and the magnitude of random and systematic errors should be budgeted carefully [6].

Various articles discuss topographic Lidar QC measures (e.g., [7,8,9]); however, only a limited number note ALB methods and discuss comparisons with other relevant technologies, providing recommendations (e.g., [10,11,12]). It is critical to understand and quantify the expected ALB survey accuracies, which rely heavily on environmental conditions and specific system capabilities. The International Hydrographic Organization (IHO) defines Order 1(a) standards (for waters shallower than 100 m) and states maximum allowed total vertical uncertainty (TVU) at 2-σ confidence requirement [13]. Current ALB systems in the market cannot achieve depths to 100 m; however, they exceed the Order 1(a) accuracy requirements for depths less than 10 m [5].

Overall, bathymetric surveys allow us to measure the depth of water bodies and understand the hydrologic and geologic properties of individual basins to support various applications such as contour modeling, flood inundation, and leakage. A number of bathymetric river surveys were completed in recent years using ALB technology (e.g., [14,15,16,17,18,19]); and these studies demonstrated the practice as a viable and detailed method for shallow-water mapping. Because the geomorphology of rivers varies, the success of an ALB survey relies on several environmental aspects and conditions, especially if the survey location includes riparian areas with overhanging trees and submerged vegetation in the water column. Earlier studies demonstrated enhanced modeling and filtering, and provided guidance to optimize modeling methods with potentially difficult river surveys [12,17,20]. In this project, throughout the survey location, we identified locations with substantial water storage (basins) and approached certain areas with multiple flight lines and different headings, to minimize the shadow effect caused by overhanging vegetation. We conducted several supplementary surveys and applied relevant QC measures to demonstrate the effectiveness of using ALB technology to characterize the complex geomorphology of a river with a densely vegetated riparian and aquatic zone.

## 2. Materials and Methods

### 2.1. Study Area

**Figure 3:**
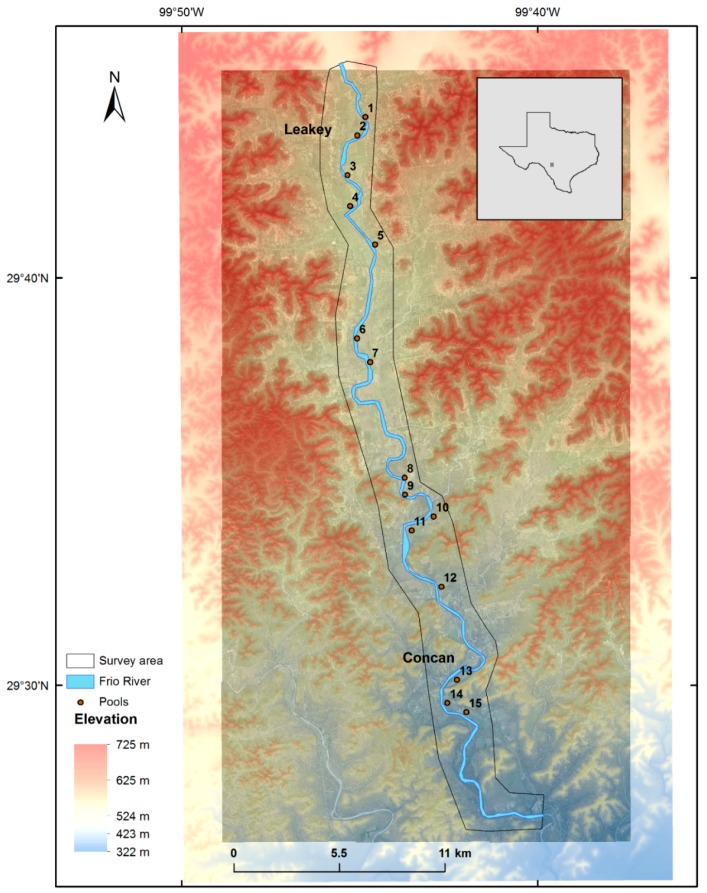
Frio River study area. Basins are in sequence from one to fifteen, north to south.

**Figure 4:**
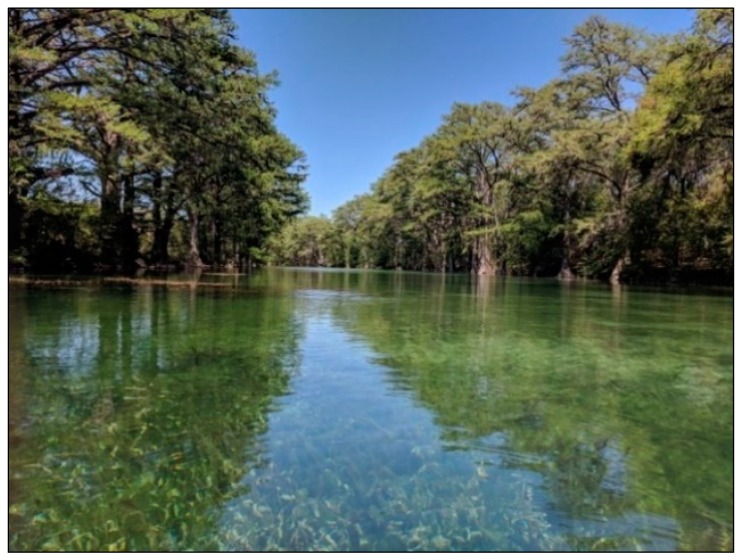
Frio River and its scenic environment, surrounded by overhanging trees along the riparian zone.

**Figure 5:**
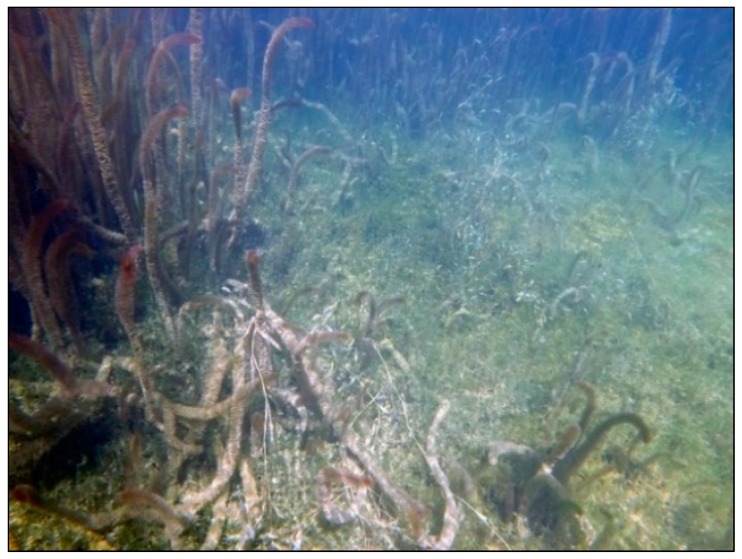
Diverse submerged assemblage, which creates challenges for Lidar and sonar measurements.

Frio River begins in northeast Real County, Texas, and flows in a southeasterly direction for about 400 km, traversing Uvalde, Medina, Frio, La Salle, McMullen, and Live Oak counties, where northern sections of the river wind through the scenic Frio Canyon in the Texas Hill Country. The river passes through limestone canyons lined with a variety of tree species. Upper sections of the river, north of U.S. Route 90, are picturesque; overhanging trees provide a shaded setting for recreational activities and help to maintain an ecologically rich and diverse environment. The aquatic and riparian habitats of the Frio River support an exceptionally diverse assemblage of invertebrates, fish, birds, weeds, and plants of the Edwards Plateau [21]. Figure 3 presents the topography of the survey location and 15 basins with substantial water storage as identified, in sequence from north to south. Field staff had access to some of these basins with a custom-built kayak and were able to complete supplementary surveys for QC purposes (Figure 4 and Figure 5).

Numerous springs along the river provide valuable recharge along the outcrop portion of the Edwards Aquifer. The river ultimately empties into the Nueces River, contributing freshwater inflow to Nueces and Corpus Christi bays on the central Texas Gulf Coast. Because of recent drought conditions, the U.S. Geological Survey (USGS) gage (08195000, Frio River at Concan, Texas) reported less discharge and lower gage height levels at lower sections of the river. For the month of April, since 1925, USGS gage recorded mean monthly discharge rate of 2.6 m^3^/s; however, in April 2018, the mean discharge rate was less than 1 m^3^/s. USGS also reported a gage height at 1.06 m, which is 7 cm lower than the average of the previous 19 years of measurements. Such lower water levels are an environmental concern; however, these conditions contributed to possibly lower turbidity levels and shallower water-column depths—an advantage for this particular ALB survey.

### 2.2. Ground Control

Acquiring precise ground-control data with GNSS receivers during ALB surveys is critical. The quality of these data may directly affect final product accuracy. In the US, the public can access these data sets online at a number of already established stations. Continuously Operating Reference System (CORS) is an active network, operated by local agencies and overseen by the National Oceanic and Atmospheric Administration (NOAA). Some of the more than 2000 stations nationwide can have custom sampling rates (e.g., 1, 5, 10 s) with requests to their maintaining agency. We accessed TxDOT-maintained TXUV (Texas Uvalde) and TXLE (Texas Leakey) stations, sampling data at 1-s intervals. Table 1 illustrates the accuracy findings of TXUV and TXLE stations at all directions (latitude, longitude and elevation), as measured and averaged in 2018. We measured the mean *Z* accuracy at less than 1 cm.

### 2.3. Lidar System Calibration

Because the Chiroptera’s NIR- and green-wavelength scanners function independently, it is important to understand and minimize common offset errors in order to have seamless data sets. Positional information and accuracy of the Lidar returns are the result of combining the derived measurements of system components and specific mounting parameters, and these are reported by the system manufacturer. All Lidar systems currently available on the market are equipped with manufacturer provided applications that optimize boresight angles. However, we recommend Lidar analysts complete a manual and visual verification by overlaying Lidar strips from NIR and green wavelength scanners, and verify calibration by checking man-made and natural features on the ground (e.g., trees, slanted roofs, highway bridges). For interested readers, several articles describe the mathematical relationship and the importance of correct boresight angles (e.g., [6,8,22]). Shin et al. [23] discusses synchronization problems between different scanners for ALB surveys.

For the Frio River project, we acquired Lidar data using NIR and green wavelength scanners at a 500-m range over the Del Rio International Airport (DRT), in opposite and adjacent directions, and used Leica Lidar Survey Suite v2.4 (LSS) to compute and optimize roll, pitch, and yaw angles (*R*_Δω,ΔΦ,ΔΚ_). For slant-range correction (vertical bias), we acquired 182 ground-control points over the taxiway surface using a geodetic level GPS receiver (R8 GNSS, Trimble, Sunnyvale, CA, USA). Because Lidar points do not coincide and overlap, and we assume no redundancy with Lidar- and GPS-derived data sets, we cannot assume 1:1 comparison between individual Lidar returns and GPS points. Therefore, we generated planar patches that represent the mean elevation of a number of Lidar returns in the close proximity and compared those mean elevations to the control points. We used the least-squares statistical approach and calculated the goodness-of-fit, and observed the residuals. *R*^2^ value represents the closeness of data and the robustness of adjustments, which is the percentage of the response-variable, explained by an independent variable. Additionally, we computed the root-mean-square error (RMSE) to identify the differences between values predicted by a model and values observed.

### 2.4. Airborne Missions and Data Acquisition

A total of 101 individual flight lines covered an area of 80 km^2^. The flight crew spent approximately 15 h in the air, acquiring Lidar data from both scanners concurrently with high-resolution imagery. Average ground speed was 120 knots per hour. Over flat areas, the NIR Lidar data sampling averaged up to 26 points/m^2^, and bathymetric returns averaged 1.65 points/m^2^. Output of all point-cloud data resulted in approximately 5 billion points. For ease of data processing and analysis, we divided the survey area into 1-km^2^ blocks and separated Lidar data sets into respective classes.

Flight lines were separated from each other by 160–180 m, achieving an average of 40 percent overlap and a ground-swath width of 250 m. The overlap was a critical planning consideration, given that ground altitude of the survey location greatly varied; any lesser amount of overlap would have potentially created gaps between the strips as the swath narrowed at smaller ranges. Because of abrupt changes in the elevation of the terrain in the southern sections of the river, we experienced the “multiple pulses in the air” (MPIA) phenomenon while collecting NIR data in the area. The affected flight lines were re-flown at the same altitude, with the system configured to use a slightly lower repetition rate (240 versus 260 KHz), which provided MPIA-free data. In order to prevent the need for repeated flights, we recommend using flight-planning applications very carefully, with consideration given to the limitations imposed by their particular system. Staff should manually verify the selected parameters within acceptable limits at all locations, especially at high or low terrain areas. In addition, some ALS systems include hardware and software features on the sensor that help to compensate for variable terrain elevation, such as the ability to detect and correct MPIA on-the-fly or adjust field of view, which can help to eliminate gaps caused by variable elevation. Nevertheless, performing a manual check of the terrain would ensure that the selected flight and scanner parameters do not exceed the system’s operating limitations. In order to avoid MPIA, range (*r*) and pulse repetition rate (*p*) should be carefully selected. Equation (1) defines the mathematical relationship with *C* = speed of light and (*s*) as the fractional margin of safety:

(1) $$r=\left(\frac{C}{2\times p}\right)\times \left(1-s\right)$$

### 2.5. Waveform Analysis

Waveforms, with their potential to provide information about water condition and bottom characteristics of a water body, are a valuable source of data. In simple terms, an ideal waveform is composed of two distinctive peaks, representing returns from the water-surface and the bottom. The number of samples (time signals), slope, and amplitude of backscatter provide information about the water depth, diffuse attenuation, and bottom properties. In slow-flowing rivers and still lakes, where water is mostly calm, transparent and shallow, NIR reflections from the surface can be very weak and difficult to detect, and the green wavelength laser might not detect the surface at all. In such cases, the LSS surface-detection algorithm builds an interpolated surface, using all available returns from both scanners. Because of such uncertainties, bathymetric waveform analysis is an active area of research; several recent studies are available to the interested reader. Eren et al. [24] discussed a novel approach to identify water-bottom characteristics by studying backscatter amplitude. Richter et al. [25] examined the diffuse attenuation in the water column and presented an experimental approach for correcting turbidity effects. Zhao et al. [26] proposed a method of decomposing raw waveforms; using Gaussian, triangle and Weibull functions.

Chiroptera samples up to 1024 times in a single waveform, but we suggest acquiring data with a lesser sampling rate because of storage issues, especially for larger surveys. Binary information can be exported into text format. We further analyze the data using MATLAB v17, which is a commercial application package but other statistical applications (e.g., R, GNU Octave) are free of charge. Using an in-house built algorithm, it is possible to compute depth by calculating total discrete time signals (*Δt*) between the backscattering surface and bottom peaks (*t*_1_ and *t*_2_). The equation requires the refraction index value as the only variable (*n*), which relies on water temperature, salinity, and the specific light wavelength. Chiroptera uses 515 nm, specifically. Speed of light and digitizer sampling frequency (*f_s_* = 1.8 GHz) are constant values to compute the backscatter travel distance (Equation (2)):

(2) $$d_{m}=\frac{C\;\Delta t}{2nf_{s}}$$

### 2.6. Sonar Survey

**Figure 6:**
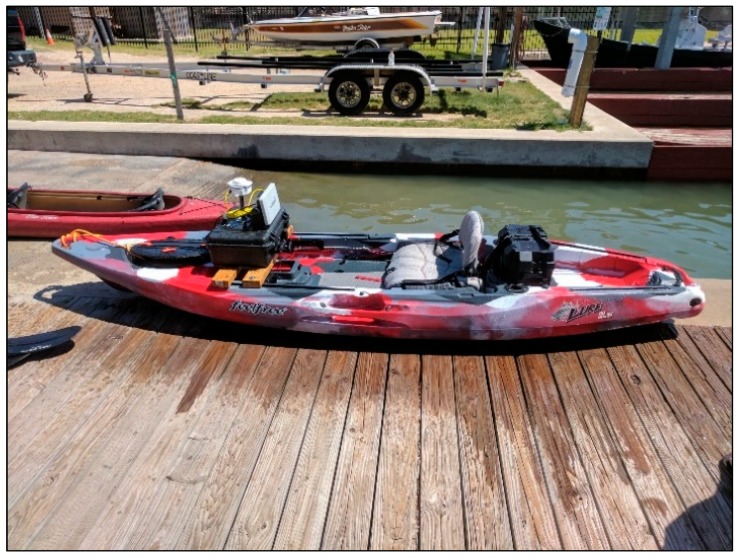
Kayak with sonar equipment.

In shallow and transparent waters, waveform information will indicate the surface and bottom positions with strong peaks; however, these may become less significant and difficult to distinguish in very shallow or wavy waters [27], appearing as noise-like. Furthermore, various other environmental conditions may influence the outcome of an ALB survey, making supplemental surveys beneficial or necessary [5]. In such conditions, sonar surveys may provide essential depth information, as well as additional means to confirm the Lidar measurements [28]. In the Frio River, we had access to 8 of 15 basins for sonar surveys, and measurements were focused on sections with overhanging riparian vegetation potentially blocking the laser. A custom-built kayak with a battery-powered trolling engine was an ideal platform because of its mobility and capability of maneuvering in tight and shallow areas of the river (Figure 6).

**Figure 7:**
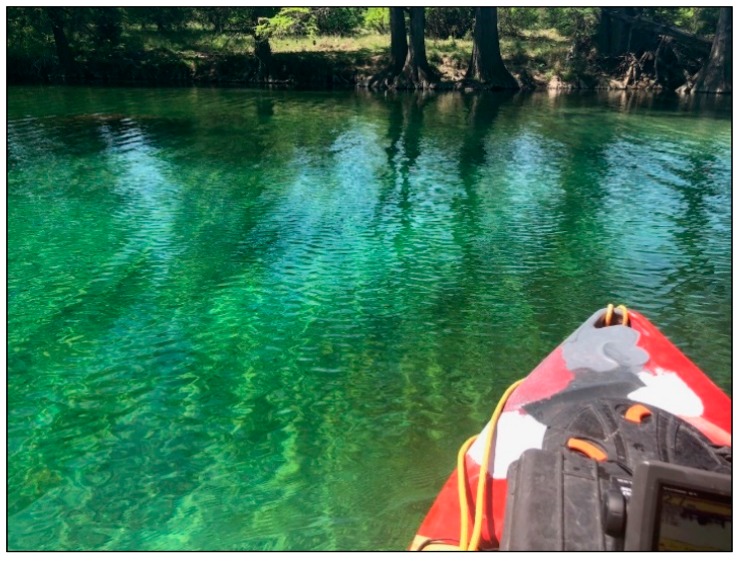
Calm and shallow waters of the Frio River.

**Figure 8:**
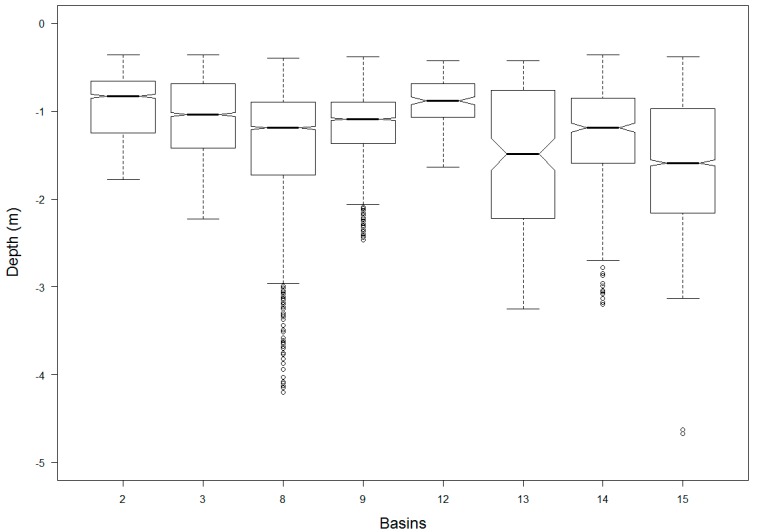
Sonar measurements in the Frio River survey. Basin #15 is deepest with a mean depth of 1.58 m and basin #12 is shallowest at 0.91 m.

The sonar unit, a Garmin EchoMap 74dv, features a multiband sonar transducer (GT21-TM, Garmin, Olathe, KS, USA) with an effective depth range of 36 cm to 700 m in freshwater bodies. It also samples positional data at a rate of 5 Hz with a built-in GPS interface. In the lab environment, we tested the unit and observed a standard deviation of 2.7 cm at 11.8 m depth. We further investigated the sonar for its positional accuracy, without applying any corrections. Of all positions, mean offset was less than 1 m for longitude, and ~2 m for latitude from a control point, which is adequate for basic positional information purposes in slow flowing river conditions (Figure 7). Because the Frio River is shallow and mostly calm, we did not consider rotational angle adjustments for roll, pitch, and yaw information with sonar measurements. However, for deeper, faster flowing and choppier water-body surveys, especially marine shoreline surveys, we suggest considering rotational computations for higher positional accuracy. Of all 8-basin surveys, we measured 0.36 m as the shallowest and 4.67 m as the deepest water column using the sonar. Mean depth was calculated at 1.29 m (Figure 8).

### 2.7. Water-Surface and Bottom Confirmation

We measured water-surface and bottom elevations at designated locations throughout the river using a geodetic-grade GNSS receiver (Trimble R8) attached to a 2-m pole. As a practice, measuring water-surface is difficult; therefore, we surveyed representative locations at the water’s edge. For bottom measurements, we used rock or hard surfaces to prevent the survey rod from sinking into the bottom.

Water-surface measurements are generally required to confirm the accuracy of ALB-derived surfaces and should be concurrent with airborne missions. At inland waters, minor variations may be expected because of ripples and flow conditions. With shoreline surveys, tidal effects should be considered; lower tides are preferred for scheduling airborne missions. At shallow areas, one can estimate the depth by using a simple measuring device (e.g., marked rod with bubble level, or string with weight), but such measurements require positional information and may not be accurate at deeper and faster-flowing conditions. At remote locations with no in situ measurement access, satellite bathymetry may be an option for estimating the depth of larger lakes and shallow coastlines, albeit with various considerations such as ground resolution, time of capture, and water transparency [29,30], however, this approach is not adequate to validate Lidar bathymetry accuracy. An unmanned airborne vehicle (UAV) with a tethered sonar may provide good bathymetry, but such surveys are localized, restricted with dense aquatic vegetation, and difficult to navigate at locations with heavy riparian vegetation [31].

### 2.8. Water-Transparency Measurements

**Figure 9:**
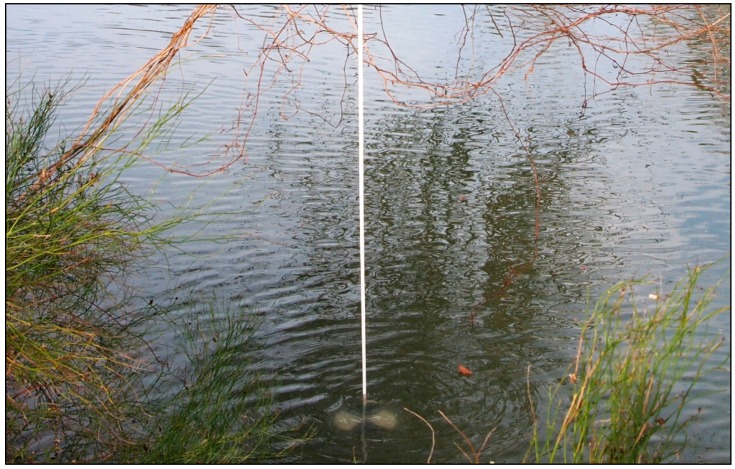
Freshwater Secchi disk.

Water transparency and predicting its effects on Lidar bathymetry is a challenging task for an ALB survey. Because organic and other suspended material in the water scatters and attenuates light-beam energy, describing water-column conditions in quantitative terms is a good practice. Instead of specified requirements, ALB surveys have advised values indicating transparency and turbidity. The Secchi depth, which indicates the maximum depth at which a black-and-white circular disk (Figure 9) can be seen when progressively lowered into a water body, is not a definite predictor of ALB survey performance, but rather a surrogate to calculating the diffuse attenuation coefficient [32]. ALB systems vary with their expectant depth performance, and AHAB expects Chiroptera to measure depths at 1.5 times the Secchi depth when benthic zone reflectance conditions are greater than 15 percent. A number of empirical relationships have been developed between Secchi depth (*Z_SD_*) and diffuse attenuation (*K_D_*, m^−1^) based on measurements in various water bodies, therefore, a varying coefficient value (*α* = *K_D_* × *Z_SD_*) has been reported in the range of ~ 1.3 to 2 [33]. A more quantitative and prevailing approach would include an empirical assessment of inherent optical properties of water column using a hyperspectral imaging system [34]. Interested readers can refer to articles that provide further information and discuss the effects of diffuse attenuation on ALB performance (e.g., [35,36,37]).

**Figure 10:**
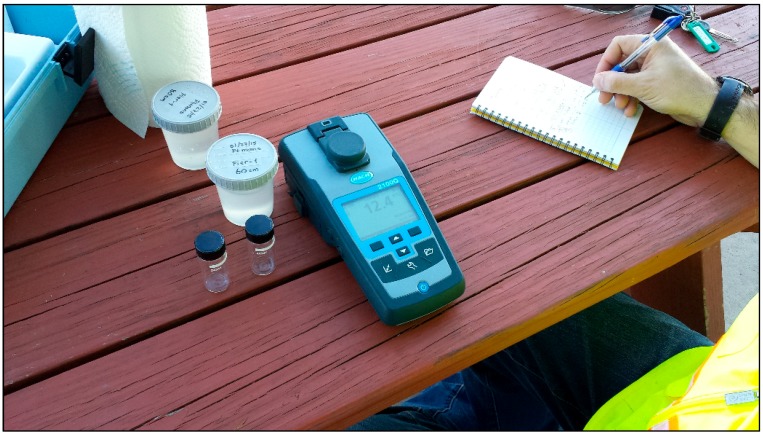
Hach 2100Q field turbidimeter.

Turbidity is an expression of the optical property that causes light to be scattered and absorbed rather than transmitted in straight lines through the sample [38,39]. Expressed in nephelometric turbidity units (NTU) in the U.S., turbidity causes light to scatter in the water column, where particle size, shape, and composition may affect its amplitude [40,41]. In this project, we measured turbidity with a Hach 2100Q, a portable field turbidimeter that complies with Environmental Protection Agency (EPA) method 180.11 and produces results between 0 and 1000 NTU [42]. As part of QC compliance, all turbidimeters require calibration verification before and after each measuring session with factory-sealed samples. In the Frio River, a calibration check with predefined samples indicated a better-than-95-percent accurate reading of all samples (Figure 10).

In the Frio River project, we conducted turbidity and diffuse attenuation measurements throughout the river at selected locations. Researchers should be aware of Secchi disk measuring difficulties at fast-flowing river conditions where an exact location reading may not be possible. Because the river was shallow and calm, we observed the Secchi disk at the water-bottom as visible at all locations and expected Lidar beams to map the bottom.

## 3. Findings and Discussion

### 3.1. System Calibration

**Figure 11:**
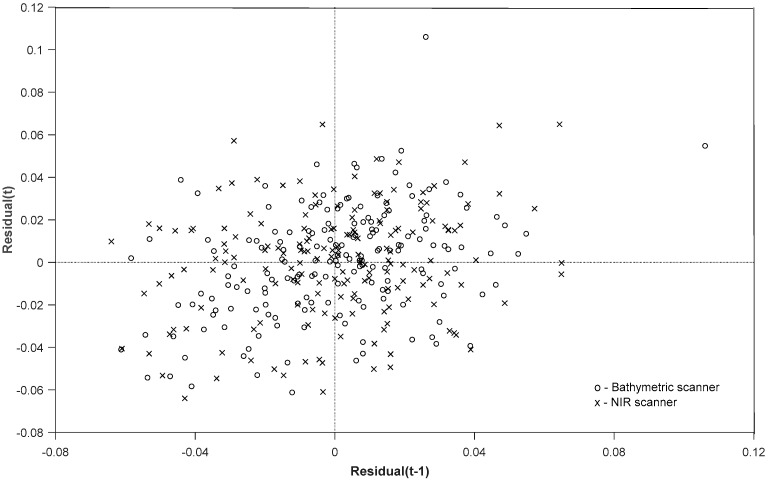
Slant-range adjustment of Chiroptera scanners; control points versus Lidar-derived patch elevation-lagged residuals. Residuals do not indicate any underlying patterns, but only a few outlier measurements.

We compared 174 ground-control points to Lidar planar-patch elevations, averaged by the returns in a 2-m vicinity of a control point. In the calibration process, elevation heights calculated are ellipsoidal and true to GPS and Lidar data acquisition, so there are no transformations applied. We converted the ellipsoidal elevations to orthometric height (real-world elevations) using the GEOID2012B model to produce the final products. Results indicated a good fit for both scanners; median bias was less than 2 cm, and we calculated the RMSE at less than 3 cm (Table 2). Figure 11 illustrates the lag plot of both scanners and checks whether a data set is randomly distributed with no underlying common patterns.

**Figure 12:**
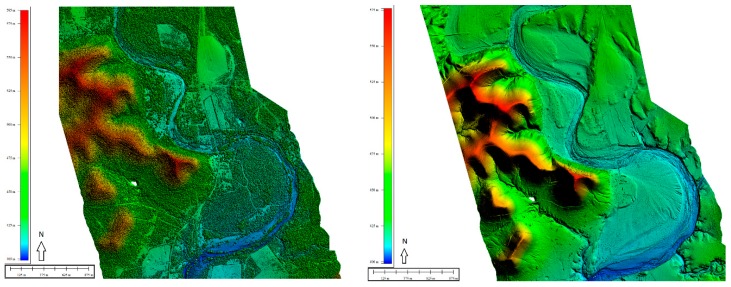
Garner State Park area, Texas, surface and elevation models of the Frio River and its surroundings.

Additionally, we analyzed the quality of GPS post-processing results. Because we acquired static data to measure water-surface and bottom, refining the horizontal and vertical accuracies was necessary. Understanding the possible positional biases caused by surrounding tall trees and high cliffs—increased high dilution of precision value—is vital, especially at some locations (e.g., Basin 9 by Garner State Park, Figure 12). Post-processing results revealed slightly high minimum versus maximum (range) values but confirmed low standard-deviation values for vertical measurements at smaller than 2 cm (Table 3).

### 3.2. Water-Bottom Confirmation

**Figure 13:**
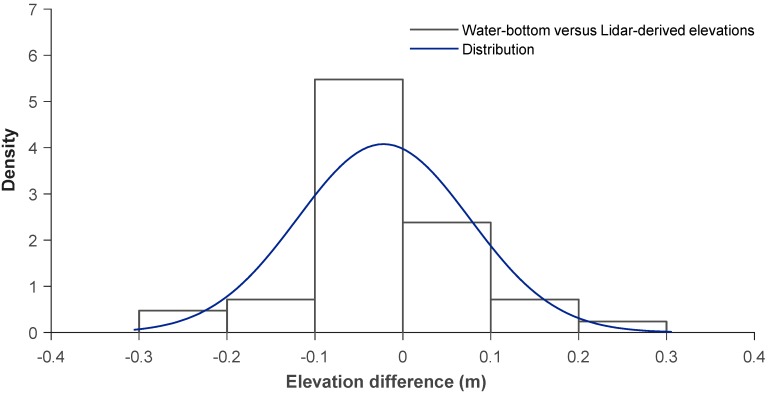
Distribution of GPS measured water-bottom elevations to Lidar-derived elevations.

We concluded 40 water-bottom measurements using a GPS, and correlated findings to bathymetric Lidar-derived elevations. We used the Delaunay algorithm to build a triangulated irregular network of planar patches of Lidar returns that register within 2 m of a control point, at slopes less than 30-degree. In our equation, we subtracted Lidar-derived elevations (*h*_L_) from differentially corrected GPS (*h_g_*) measurements to calculate the height difference (*d*_Z_ = *h*_L_ − *h*_g_). Results showed a good correlation and a normal distribution; although Lidar-derived elevations were slightly lower (Figure 13).

Mean error was −0.01 m and we computed standard deviation at 0.08 m. As an average, adjusted *R*^2^ = 0.929 indicated a very robust correlation (Table 4).

### 3.3. Water-Surface Confirmation

**Figure 14:**
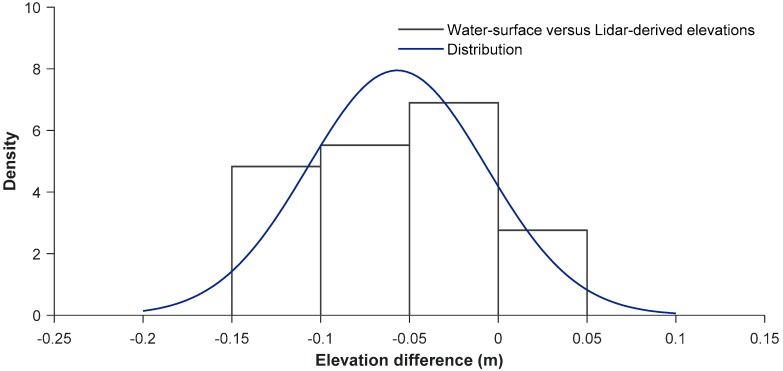
Distribution of water-surface elevation to Lidar-derived surface returns.

We measured water-surface at 28 locations using a GPS, mostly at the water’s edge, and differentially corrected the measurements. Because the Frio River was shallow and transparent, green wavelength returns did not entirely detect the surface, and returns were scattered. LSS simulated the surface by using all available returns, from both scanners, so we expected minor variations in the data range. We subtracted the Lidar-derived patch elevations from GPS measurements, and averaged the results (Table 5). Of all basins, we computed a standard deviation of 0.05 m, and mean difference was calculated at −0.06 m (Figure 14), where Lidar-derived elevations were lower (394.94 m versus 394.88 m).

Researchers surveying at similar water and environmental conditions are encouraged to complete in situ surface measurements. Considering the high accuracy achieved with water-bottom measurements, surface measurement findings are slightly less accurate and might have an influence on specific applications such as water-shed management and flood basin mapping.

### 3.4. Turbidity Analysis

We sampled water conditions at selected locations throughout the river and found turbidity levels to be low, which is ideal for ALB surveys. Overall, lower turbidity levels scatter the light beams less and contribute to stronger backscatter amplitude at the receiver. In each sampling location, we conducted three measurements and recorded the median readings. Standard deviation was less than 0.15 NTU for all readings (Table 6).

Results indicated an overall turbidity level less than 1 NTU and water-column depth shallower than 3.2 m. Under such conditions, diffuse attenuation and scattering is low, and we expect Lidar beams to measure the bottom with sufficient backscatter amplitude.

### 3.5. Waveform Analysis

To confirm the sonar and LSS-derived depth readings at selected turbidity-measurement locations, we extracted and analyzed waveform information. Using LSS, we created 5 × 5 m blocks at selected sampling locations and exported the information into MATLAB (v17). Each time signal (sample) corresponds to an estimated depth of 6 cm with Chiroptera’s current configuration.

**Figure 15:**
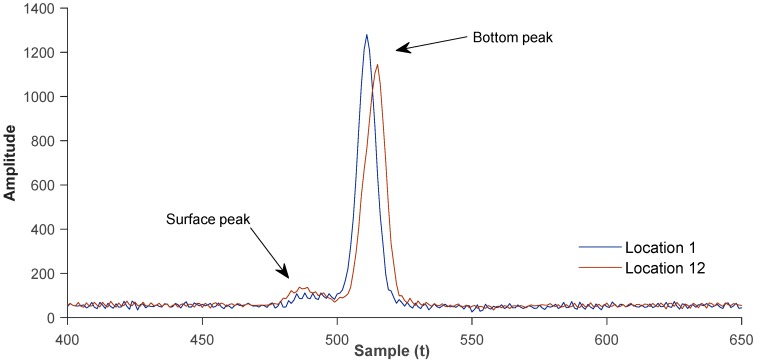
Averaged waveforms at sampling locations 1 and 12. Surface peak is weak, and bottom backscatter is strong, indicating shallow and transparent water columns.

At sampling location 1 (Basin 2), water was shallower and reflectance from the gravel bottom layer was higher, resulting in greater backscatter amplitude. We sampled and averaged 22 waveforms, computing 25 samples between peaks. We computed the depth at 1.56 m, and the finding indicated a 16-percent-deeper measurement compared to sonar depth. At location 12 (Basin 9), submerged vegetation and sand bottom caused a less reflective environment, producing less backscatter amplitude; however, a more distinctive surface peak and a slope (between the peaks) was evident. We sampled and averaged 17 waveforms, computing 29 samples between peaks. We concluded the depth at 1.81 m, where water column was 9-percent-shallower compared to sonar, possibly caused by a number of wider Lidar pulses returning from submerged weeds (Figure 15).

For both locations, waveform analysis indicated a weak surface peak and a strong reflection from the bottom, which is an expected response for shallow and transparent water columns. In deeper and possibly less-transparent waters, we would expect the peaks as reversed: surface peak amplitude is greater than bottom peak because of energy loss and scattering in the water column.

### 3.6. Sonar Measurements

We evaluated 10,600 sonar measurements in conjunction with the airborne missions. Scheduling airborne and sonar surveys at the same time to ensure similar environmental conditions was critical for the mission. For QC analysis, we created planar patches from bathymetric Lidar returns and correlated the mean elevation to sonar-derived elevations at selected basins using least-squares analysis. Findings indicated a mean error of 13 cm and standard deviation of 30 cm (Table 7).

**Figure 16:**
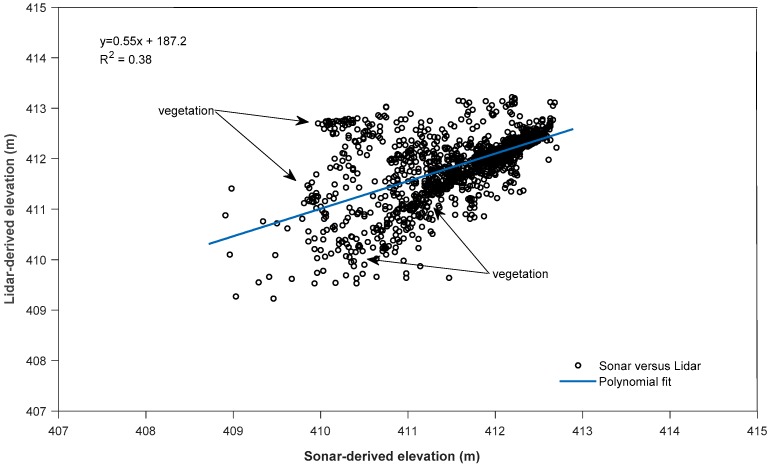
Basin 9 Lidar-derived elevation versus sonar-derived elevation—densely submerged vegetation in deeper areas.

**Figure 17:**
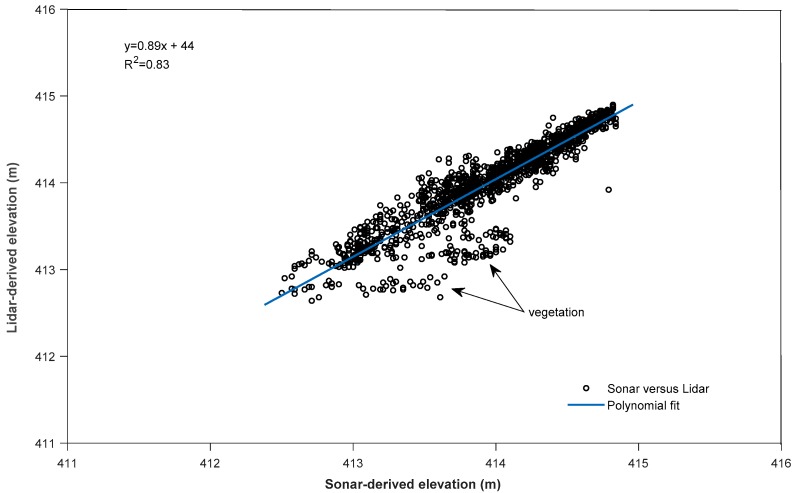
Basin 8 Lidar-derived elevation versus sonar-derived elevation—moderate amounts of submerged vegetation in water column.

Because the sonar pulse beam is much narrower, it registered greater water depths at certain areas, especially in basins with dense submerged vegetation. This phenomenon was mostly evident in Basin 9, where Lidar signals did not freely enter the water column because of dense submerged aquatic vegetation, resulting in highest data sampling range and poor correlation at depths of 2 m and greater (Figure 16). Basin 8 findings indicated the lowest mean error and a good probability that Lidar-derived elevation and sonar-derived elevation matched each other with moderately submerged vegetation (Figure 17).

## 4. Conclusions

This article summarizes quality-control measures applicable to ALB river surveys as an example, and describes the efforts conducted for the Frio River survey in Texas. Understanding the accuracy of final products is essential for all ALB surveys, and we emphasize the importance of supplemental surveys for quantifying the quality of bathymetric- and topographic-data sets. The practices discussed in this article are intended to be general so that other organizations can establish their own measures and practices and confirm the results by other possible means in accordance with project requirements. In the Frio River survey, the environmental conditions were ideal for a successful Lidar survey. At other locations with deeper and less transparent water column conditions, results would vary. However, the positive outcome was also a product of well-established quality-management and QC procedures and expertise gained over the years. We believe the following considerations are valuable for similar future deployments:

- Sonar and GPS surveys are an indispensable component of ALB projects and provide the optimum means for understanding the quality of Lidar bathymetry products.
- Understanding water transparency in quantitative terms is vital for success of ALB surveys, especially in deeper and more turbid conditions.
- Waveform information provides the means to confirm depth findings and may provide supplementary evidence about water-column and water-bottom characteristics where in situ measurements are not feasible or possible.

Multiband satellite imagery, unmanned survey boats, and aerial vehicles may provide very valuable supplemental information and contribute to minimizing ALB uncertainties.

## Figures and Tables

**Table 1:** CORS stations TXUV and TXLE, and positional accuracies as reported in 2018.

Reference Station	Latitude	Longitude	Ellipsoidal Height (H, m)	Mean Accuracy (N, cm)	Mean Accuracy (E, cm)	Mean Accuracy (Z, cm)
TXUV	29°12′ 01.86729	99°49′ 37.57853	262.570	0.07	0.04	0.84
TXLE	29°44′ 21.58283	99°45′ 40.5039	478.402	1.57	−0.01	−0.41

**Table 2:** Vertical-bias adjustment results after Chiroptera system calibration.

Lidar Scanner	Number of Samples	Sample Range (m)	Median (m)	RMSE (m)	Adjusted R^2^
NIR	174	0.13	0.004	0.026	0.998
Green-wavelength	174	0.16	0.016	0.026	0.999

**Table 3:** GPS post-processing quality-control findings (vertical adjustment).

Measurement	Number of Samples	Sample Range (m)	Median (m)	SD (m)
Water-surface	28	0.11	0.04	0.02
Water-bottom	40	0.08	0.05	0.01

**Table 4:** Comparison of water-bottom measurements to Lidar-derived elevations (*d*_Z_ = *h*_L_ − *h*_g_).

Basin	Number of Samples	Mean Lidar-Derived Basin Depth (m)	Mean Difference (m)	RMSE (m)	Adjusted R^2^
2	14	0.99	−0.01	0.066	0.958
3	11	1.02	−0.01	0.063	0.967
9	8	0.72	−0.03	0.055	0.915
12	7	1.36	−0.01	0.125	0.878

**Table 5:** Water-surface measurement comparison to Lidar-derived surface returns (*d*_Z_ = *h*_L_ − *h*_g_).

Basin	Number of Samples	Sample Range (m)	Mean Difference (m)	SD (m)	RMSE (m)
2	8	0.06	−0.09	0.02	0.02
8	6	0.12	−0.03	0.02	0.02
9	2	0.16	−0.10	0.04	N/A
12	4	0.07	−0.01	0.04	0.05
15	8	0.15	−0.04	0.06	0.03

**Table 6:** Turbidity and depth measurements at selected locations in the Frio River.

Sample	Basin	Location (Latitude, Longitude)	NTU (Median)	SD (m)	Sonar Depth (m)
1	2	29.724465, −99.749098	0.42	0.02	1.40
2	3	29.708657, −99.756977	0.66	0.15	1.38
3	3	29.706622, −99.755463	0.43	0.02	2.09
4	12	29.542181, −99.714676	0.44	0.03	1.73
5	12	29.538870, −99.713520	0.48	0.05	1.35
6	15	29.488645, −99.706318	0.59	0.03	2.06
7	15	29.488227, −99.702751	0.57	0.12	2.63
8	15	29.485601, −99.698762	0.76	0.04	1.78
9	8	29.583176, −99.729285	0.55	0.04	2.10
10	8	29.579488, −99.730900	0.46	0.06	1.11
11	9	29.576960, −99.728415	0.58	0.16	3.2
12	9	29.576916, −99.727918	0.39	0.03	1.9

**Table 7:** Sonar-reading correlation results for Lidar-derived patch elevations at water-bottom.

Basin	Number of Samples	Sample Range (m)	Mean (m)	RMSE (m)	Adjusted R^2^
3	1843	2.07	0.16	0.2	0.812
8	1584	1.6	0.04	0.2	0.832
9	1840	4.56	0.23	0.48	0.382
12	2156	1.93	0.06	0.23	0.681
13	176	1.26	0.08	0.2	0.63
14	522	2.55	0.18	0.34	0.912
15	2479	3.04	0.16	0.33	0.786
